# Glyceryl trinitrate patch therapy is associated with healing of arterial ulceration and improvement in toe pressure measurements: a case study and discussion of aspects of this novel treatment modality

**DOI:** 10.1186/1757-1146-4-S1-P36

**Published:** 2011-05-20

**Authors:** Sylvia McAra

**Affiliations:** 1 School of Community Health, Podiatry Department, Charles Sturt University, Albury, NSW, 2640, Australia

## 

A case study of one frail elderly client with indolent arterial ulceration was conducted, with glyceryl trinitrate (GTN) patch therapy appearing to have a positive role in the healing outcome and improved vascular status as measured by photoplethysmography (PPG ) toe pressure measurements. The history and pharmacological action of this novel mode of therapy are briefly presented. The presentation of chilblains (erythema pernio) and arterial ulceration are discussed in the context of this case. Loss of sensitivity to cold perception, related to age, arterial insufficiency and other factors are considered. Current research using this medication for other applications suggests that GTN patch therapy should have a positive role in healing arterial ulceration associated with peripheral vascular disease (PVD). Positive outcomes with painful diabetic neuropathy have also been found with this therapy and its use warrants further investigation. A review of the literature on the use of GTN therapy for digits is briefly summarised. Important to clinical treatment of aged people with reduced vascular supply are decreased sensory functions of pain, protective sensation and temperature sensation. Further research on the effects of GTN therapy for wound healing should aim to control for seasonal climate and environmental temperature changes, as well as other factors that influence wound healing such as wound care, wound dressings and nutrition.

